# Efficacy of neuromuscular electrical stimulation as a therapy for sarcopenia in people on haemodialysis: study protocol for the STIM-HD randomised controlled trial

**DOI:** 10.1186/s13063-026-09938-3

**Published:** 2026-08-11

**Authors:** Emma L. Watson, Niamh Quann, Faizan Awan, Shaun R. Barber, Nicola Cooper, Indranil Dasgupta, Jonathan P. Folland, Laura J. Gray, Gordon McGregor, Jakob Škarabot, Meghan Tanel, Cat Taylor, James O. Burton

**Affiliations:** 1 Division of Cardiovascular Sciences, School of Medical Sciences, College of Life Sciences, University of Leicester, University Road, Leicester, LE1 7RH UK; 2 NIHR Biomedical Research Centre: Leicester, Leicester General Hospital, Gwendolen Road, Leicester, LE5 4PW UK; 3 Lived Experience Partner and STIM-HD Patient Co-Applicant, Blackburn, UK; 4 Leicester Clinical Trials Unit, College of Life Sciences, University of Leicester, University Road, Leicester, LE1 7RH UK; 5 Division of Public Health and Epidemiology, School of Medical Sciences, College of Life Sciences, University of Leicester, Leicester, UK; 6 University Hospitals Birmingham NHS Foundation Trust, Mindelsohn Way, Edgbaston, Birmingham, B15 2GW UK; 7 School of Health Sciences, University of Birmingham, Edgbaston, Birmingham, B15 2TT UK; 8 Warwick Medical School, University of Warwick, Coventry, CV4 7AL UK; 9 School of Sport, Exercise and Health Sciences, National Centre for Sport and Exercise Medicine, Loughborough University, Towers Way, Loughborough, LE11 3TU UK; 10 NIHR Applied Research Collaboration East Midlands, Leicester General Hospital, Gwendolen Road, Leicester, LE5 4PW UK; 11 Leicester British Heart Foundation Centre of Research Excellence, University of Leicester, University Road, Leicester, LE1 7RH UK; 12 University of Warwick Clinical Trials Unit, Warwick Medical School, University of Warwick, Coventry, CV4 7AL UK; 13 Institute for Cardio-Metabolic Medicine, University Hospitals Coventry and Warwickshire NHS Trust, Clifford Bridge Road, Coventry, CV2 2DX UK; 14 Research Governance Office, Research and Enterprise Division, University of Leicester, University Road, Leicester, LE1 7RH UK; 15 John Walls Renal Unit, University Hospitals of Leicester NHS Trust, Leicester General Hospital, Gwendolen Road, Leicester, LE5 4PW UK; 16 University Hospitals Coventry and Warwickshire, Clifford Bridge Road, Binley, Coventry, CV2 2DX UK

**Keywords:** Sarcopenia, Haemodialysis, Neuromuscular electrical stimulation, Randomised controlled trial, Muscle strength, Physical function

## Abstract

**Background:**

Sarcopenia is highly prevalent among adults receiving haemodialysis and is associated with reduced physical function, impaired quality of life, and increased morbidity and mortality. Although structured exercise programmes can improve muscle health, most people receiving haemodialysis are unable or unwilling to participate, highlighting the need for accessible alternatives. Neuromuscular electrical stimulation (NMES) is a non-volitional method of muscle contraction that may act as an ‘exercise mimetic’. Early studies suggest benefits for muscle strength, mass, and function; however, robust evidence from adequately powered randomised trials is lacking. The STIM-HD study aims to evaluate the clinical efficacy, cost-effectiveness, and mechanistic effects of NMES in adults with sarcopenia receiving haemodialysis.

**Methods:**

STIM-HD is a multi-centre, assessor-blind, randomised controlled trial that will recruit 228 adults receiving haemodialysis. Eligible participants must be receiving haemodialysis for at least 3 months, meet diagnostic criteria for handgrip strength, or prolonged sit to stand 5 performance. Participants will be randomised in a 1:1 manner to 12 weeks of NMES or 12 weeks of standard care. The intervention targets the quadriceps using a pre-defined protocol of high-frequency NMES, three times per week during routine dialysis. The primary outcome is the change in isometric muscle strength from baseline to 12 weeks. Secondary outcomes include muscle mass and architecture, physical function, body composition, physical activity, quality of life, safety, adherence, and healthcare resource use. Follow-up assessments will occur at 3, 6, and 9 months. A mechanistic outcomes sub-study will evaluate neuromuscular and molecular adaptations using electromyography, nerve stimulation, and analysis of skeletal muscle biopsies. Analyses will follow intention-to-treat principles and adjust for baseline values and minimisation factors.

**Discussion:**

This study will provide a comprehensive evaluation of the efficacy of NMES to be used as a treatment for sarcopenia in patients receiving haemodialysis. By integrating functional, economic, and mechanistic outcomes, STIM-HD will generate evidence to inform whether this therapy should be adopted into routine clinical care, and offer essential insights into the biological processes underpinning molecular responses to this intervention.

**Trial registration:**

ISRCTN43724916. Prospectively registered 29/11/2024.

**Supplementary Information:**

The online version contains supplementary material available at https://doi.org/10.1186/s13063-026-09938-3.

## Introduction

### Background and rationale {9a}

Chronic kidney disease (CKD) is a public health emergency that threatens to overwhelm the NHS, affecting around 14% of adults in England [1]. When kidney function drops to the point it can no longer sustain life, renal replacement therapy is required, for example haemodialysis (HD). There are currently 30,000 adults and children receiving dialysis in the UK, with this number projected to increase to 143,000 by 2033 [2]. Sarcopenia, characterised by a loss of muscle strength, mass, and function, is a frequent complication of CKD [3] affecting around 28% of people with more advanced disease [4–6]. The prevalence of sarcopenia in people receiving HD is even higher at around 70% [7]. The consequences of sarcopenia for people with kidney disease are not only related to physical disability but have also been linked to worse quality of life (QoL), depression, fracture risk, cardiovascular complications, and transplant failure, as well as with increased hospitalisation and death [8]. Therefore, strategies to maintain and even improve muscle mass are paramount to improving the health and well-being of this population.

The mainstay of treatment for sarcopenia is lifestyle modification, namely nutrition, exercise, and behavioural interventions. This helps to break the disability spiral that many patients experience where disease and deconditioning combine resulting in pronounced functional decline over time. Exercise has been shown to improve muscle mass and strength in people with sarcopenia on HD [9, 10] and national guidelines for exercise during dialysis have recently been published [11]. However, a large proportion of people on dialysis (as many as 74%) are unable or unwilling to participate in such programmes [12, 13], demonstrating that more accessible alternatives are clearly needed.

Neuromuscular electrical stimulation (NMES) is a treatment whereby the application of intermittent electrical stimuli to generate involuntary muscle contractions might serve as an ‘exercise mimetic’. This therapy is used widely in physiotherapy rehabilitation programmes [14, 15] and is now being used in clinical settings, including in intensive care units [16]. NMES could represent an ideal alternative for people on HD who are unable to exercise, therefore preventing the inevitable decline in muscle mass and strength that leads to sarcopenia. Evidence from small-scale studies has shown that NMES is safe and tolerable and can result in increased muscle strength [17–23], mass [23, 24], functional capacity [17–19, 21–24], and quality of life [17, 18], demonstrating that it may be a viable alternative to traditional exercise and may be an effective treatment for sarcopenia.

In response to this early evidence, National Institute for Health and Care Excellence (NICE) interventional procedures guidelines [IPG677] emphasised that ‘Further research should include long term, suitably powered and appropriately controlled randomised trials. Trials should report details of patient selection, and type and duration of treatment. Outcomes should include quality of life, social functioning and physiological measures’. These recommendations will be comprehensively addressed by the STIM-HD study.

### Explanation for the choice of comparator {9b}

The STIM-HD study will compare the effect of NMES on muscle strength compared to standard care. NMES is a treatment whereby the application of intermittent electrical stimuli generates involuntary muscle contractions and might serve as an ‘exercise mimetic’. This therapy is used widely in physiotherapy rehabilitation programmes [15, 16] and is now being used in clinical settings, including in intensive care units [17]. NMES could represent an ideal alternative for people on HD who are unable to exercise and therefore prevent the inevitable decline in muscle mass and strength that leads to sarcopenia. Standard care was selected as the comparator because it reflects current routine management for patients receiving dialysis. A sham comparator was not considered appropriate, as a credible NMES sham condition would necessitate delivering a minimal level of muscle stimulation, which could lead to treatment contamination.

### Objectives {10}

The overall aim of the STIM-HD study is to test the clinical efficacy of NMES as a therapy for sarcopenia in people on HD compared to standard care.

This will be achieved through the following objectives, which will all be conducted in people on HD compared to standard care:

1. To measure the effect of a 3-month (12 week) NMES programme on muscle strength
2. To measure the effect of a NMES programme on QoL and measures of physical function
3. To evaluate the cost-effectiveness of a NMES programme within an NHS costing perspective
4. To investigate if NMES results in neuromuscular adaptations and changes in muscle phenotype

## Methods: patient and public involvement, and trial design

### Patient and public involvement {11}

A national Patient and Public Involvement and Engagement (PPIE) group, comprising people receiving dialysis and their family/carers, has been established as part of other NIHR-funded research within the dialysis population. The group aims to ensure that the study’s objectives and outcomes remain relevant to patients and the public, and meets at least bi-annually.

The PPIE group contributed to the development of the research question and study design. Patients highlighted muscle weakness, central to the diagnosis of sarcopenia, as one of the most intrusive symptoms they experience. While understanding the benefits of regular exercise, many reported difficulties adhering to conventional exercise programmes. Patients considered neuromuscular electrical stimulation (NMES) a more feasible intervention for regular participation.

Based on PPIE input, several improvements were incorporated into the study protocol:

- Adjustments to enhance participant comfort (e.g. pad size and placement)
- Adaptations to the screening phase to allow a trial of NMES before consent to reduce attrition
- Input on randomisation method and contamination mitigation strategies, including clinic arrangements
- Expanded eligibility to include home haemodialysis patients

The STIM-HD study includes a PPIE co-applicant serving as a lay representative and expert patient on the Trial Management Group (TMG), and an independent, voting lay representative on the Trial Steering Committee (TSC).

### Trial design {12}

**Fig. 1:**
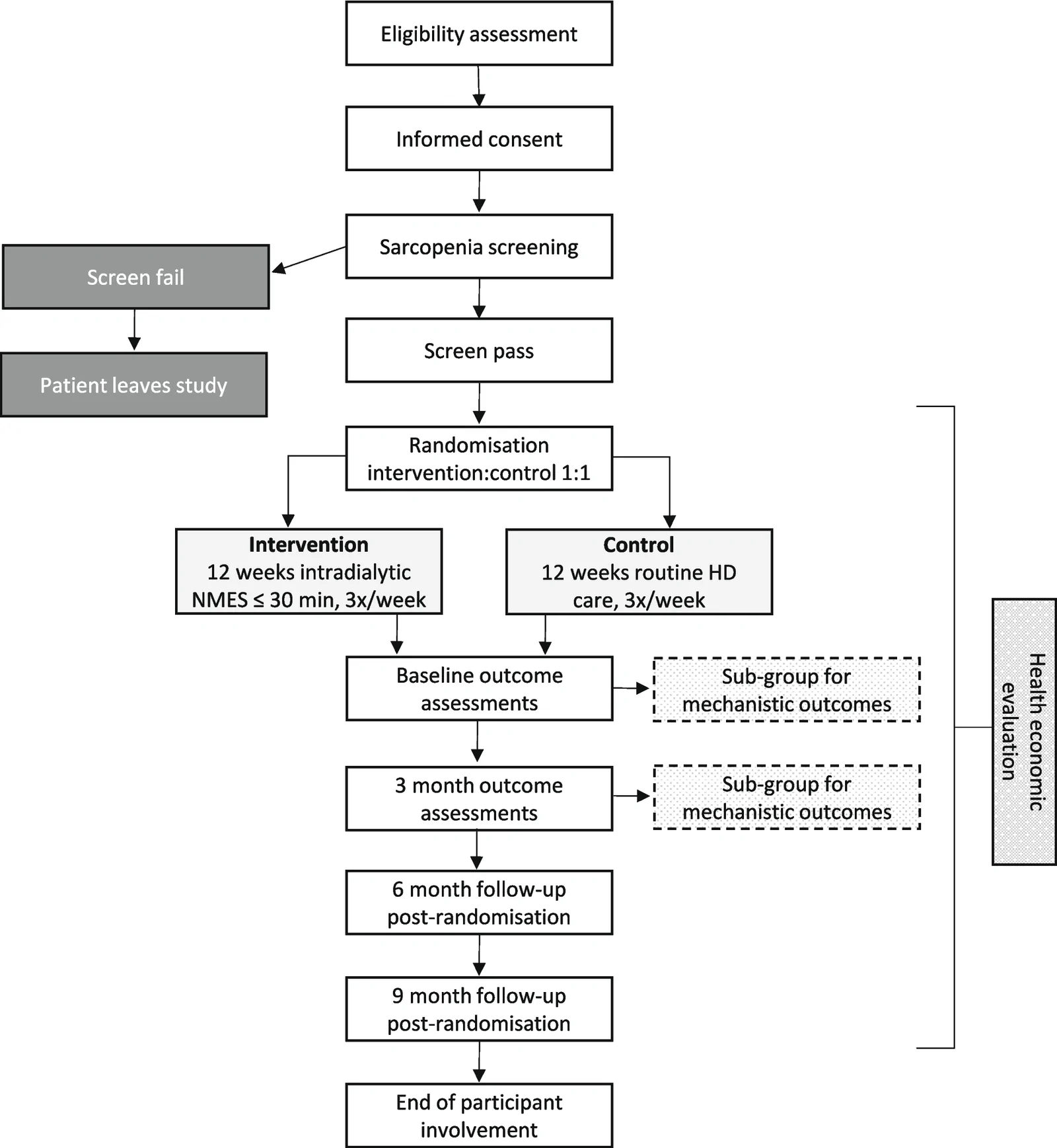
Overview of the STIM-HD study: the main randomised controlled trial, embedded health economic evaluation, and optional mechanistic outcomes sub-study

The STIM-HD study is a prospective, open-label, assessor-blind, two-arm, multi-centre, randomised controlled superiority trial with a parallel mechanistic sub-study. An evaluation of the residual 6-month impact (3 months following completion of the intervention) and a 9-month health economic analysis are embedded in this study. In total, 228 participants (114 per arm), adults with end-stage kidney disease (ESKD) and sarcopenia who are receiving long-term HD either in hospital or at home, will be recruited and randomised 1:1 to receive either 3 months (12 weeks) of NMES or standard care. Follow-up includes end of intervention analysis at 3 months with two further follow-up time points at 6 and 9 months to determine the legacy effect of the intervention (Fig. 1). In addition, 50 participants (25 in each arm) will take part in neuromuscular assessments (percutaneous nerve stimulation, and high-density surface and intramuscular electromyography), and 24 participants (12 per arm) will undergo a skeletal muscle biopsy. This study comprises 4 work packages:

- Work package 1: external pilot study to establish the optimal NMES prescription for use in the main RCT (not detailed in this manuscript and results will be published separately)
- Work package 2: main efficacy study (RCT)
- Work package 3: health economic analysis of the main study
- Work package 4: optional mechanistic outcomes study, embedded in the main study

## Methods: participants, interventions, and outcomes

### Trial setting {13}

The STIM-HD study will take place in approximately five dialysis units from up to five NHS Trusts in the East and West Midlands regions of England, with flexibility to include additional units within these Trusts if needed. The list of participating sites is available on the STIM-HD study clinical trials registration page (ISRCTN43724916).

### Characteristics of the people who are needed for the trial

**Table:** 

Characteristic	The people we would expect to see included
Age	Adults aged 18 years and older (no upper age limit)
Sex	Male, female, and intersex
Gender	Any gender
Race, ethnicity, and ancestry	White • White British • White Irish • Gypsy or Irish Traveller • Any other white background Asian or Asian British • Indian • Pakistani • Bangladeshi • Chinese • Any other Asian background Mixed or multiple ethnic groups • White and Black Caribbean • White and Black African • White and Asian • Other mixed Black/Black British/Caribbean/African • Black Caribbean • Black African • Other Black Other ethnic category • Arab • Any other ethnic background
Socioeconomic status	No specific socioeconomic criteria. Participants of any income, education, or employment status are eligible. Socioeconomic status will be recorded for descriptive purposes only as part of the resource use questionnaire
Geographic location	Study participants will be recruited in England. Study coordination will also take place in England
Other characteristics relevant to the trial	No additional participant characteristics beyond eligibility criteria are required

### Eligibility criteria for participants {14a}

The eligibility criteria for the main RCT and optional mechanistic outcomes study are reported in Table 1.

**Table 1:** Eligibility criteria for the STIM-HD study

Randomised controlled trial	
Inclusion criteria	Exclusion criteria
Undertaking HD for >3 months (home or unit based)	Contra-indications to NMES including: i. Lower limb amputation ii. Active skin ulceration or infection iii. Critical lower limb ischaemia, including pain at rest or functional impairment that precludes completion of study assessments iv. Lower limb deep vein thrombosis <3 months v. Cardiac pacemaker or implantable cardiac defibrillator
Age ≥18 years	Conditions known to cause muscle wasting i. Known neuromuscular disease ii. Active malignancy
Sarcopenia diagnosed by either: • Low handgrip strength (<27 kg for men and <16 kg for women) or • Prolonged sit to stand 5 score (>15 s for both men and women) if handgrip strength is above the sarcopenia threshold	Scheduled for living donor kidney transplant or plan to change dialysis modality or centre in the next 6 months
Able to give written informed consent	Life expectancy of <6 months
	Current participation in an interventional trial with conflicting therapies or outcomes
	Unable to give written informed consent
	Vascular access in the lower limbs that may interfere with intervention delivery
Additional eligibility criteria for muscle biopsy and intramuscular electromyography outcome measures (optional mechanistic outcomes study)	
Inclusion criteria	Exclusion criteria
As above: same inclusion criteria as the RCT	Anti-coagulation (e.g. warfarin/direct oral anticoagulant)
	Bleeding disorder (e.g. haemophilia)
	High-dose statins (i.e. atorvastatin 80 mg), growth hormone, or corticosteroids
	Deep vein thrombosis within the last 3 months

Abbreviations: *HD*, haemodialysis; *NMES*, neuromuscular electrical stimulation; *RCT*, randomised controlled trial

Once the clinical staff have confirmed that an individual is medically eligible, the research staff will obtain informed consent. Following consent, participants will undergo some additional screening assessments, including:

- Verification of sarcopenia status using handgrip strength (which must be <27 kg for men and <16 kg for women) or, if handgrip strength exceeds these thresholds, prolonged sit to stand 5 score (>15 s for both men and women).
- Evaluation of tolerance to the NMES intervention—NMES will be applied briefly to ascertain that participants can safely and comfortably tolerate it.
- Evaluation of feasibility of the strength measurements—the strength measurements, constituting the primary outcome measure, will be demonstrated to ensure participants are able to undertake them without undue difficulty.

Participants who satisfactorily complete all screening assessments will be deemed eligible for randomisation (Fig. 1).

### Eligibility criteria for sites and those delivering interventions {14b}

There are no specific eligibility criteria for sites or intervention deliverers other than completion of the mandatory training.

### Who will take informed consent? {32a}

Once a clinician has confirmed medical eligibility, informed consent will be attained by members of the study team (research assistants, research associates, PhD students, and clinicians) trained in International Council for Harmonisation—Good Clinical Practice (ICH-GCP). Informed consent will take place on the dialysis unit during potential participants’ usual HD sessions and before sarcopenia screening is performed.

### Additional consent provisions for collection and use of participant data and biological specimens {32b}

The optional mechanistic outcomes study will collect skeletal muscle biopsies and blood samples for research purposes. Participation in these procedures is optional and individuals can decline to participate, but may still participate in the main RCT. Participants will be asked to provide informed consent for any remaining samples to be retained in a research tissue bank for use in future ethically approved research.

## Intervention and comparator

### Intervention and comparator description {15a}

**Fig. 2:**
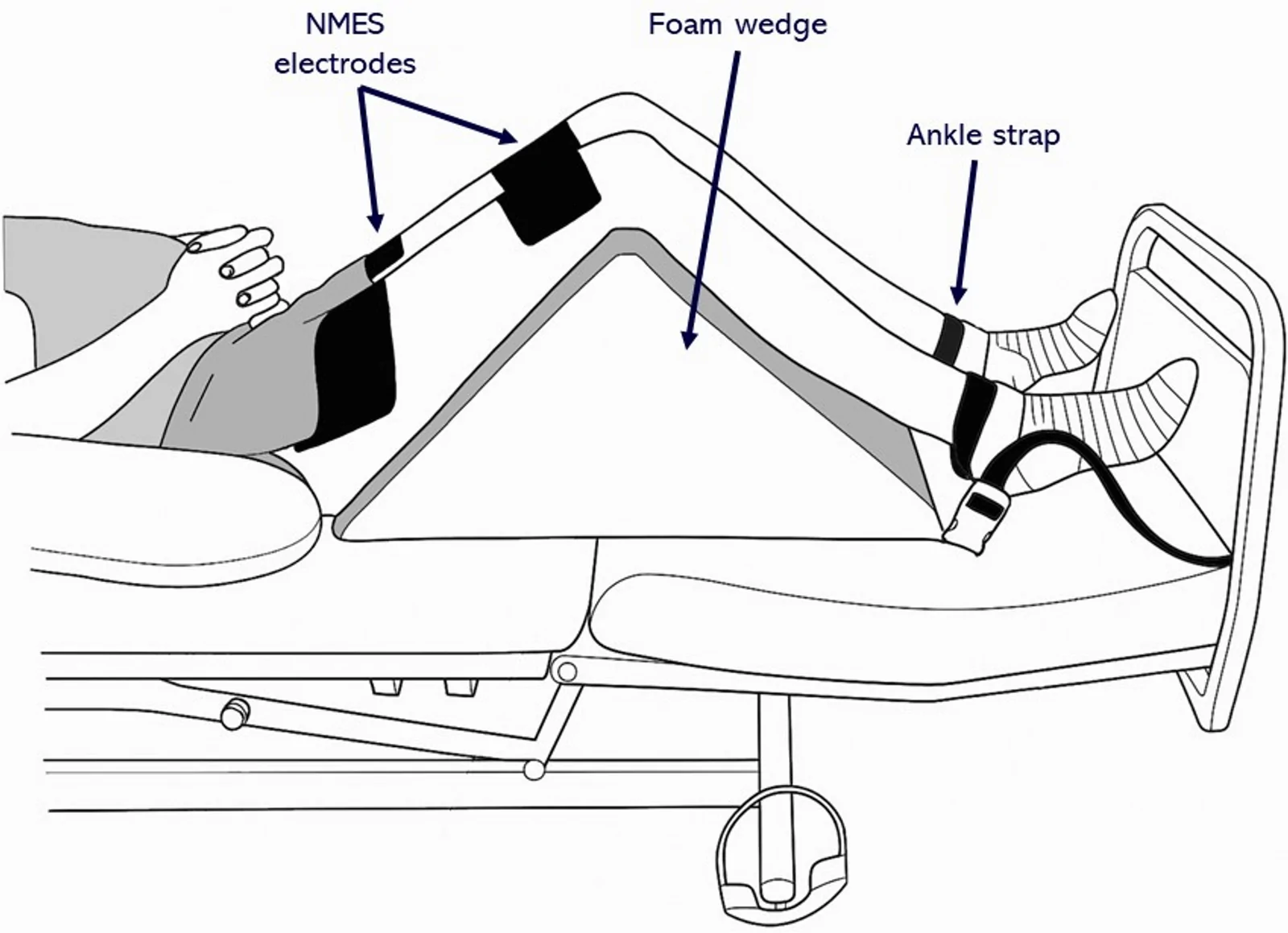
Representative image of NMES intervention delivery (image shows anatomical placement of NMES electrode pads, foam wedge, and ankle strap in a patient semi-reclined in a dialysis bed)

Participants randomised to the intervention group will receive NMES at each dialysis session they receive for 3 months (three times per week for 12 weeks). NMES will be administered to the quadriceps muscle group of both legs using a 4-channel transcutaneous electrical nerve stimulation (TENS) machine (EV906B, MedFit, UK) and two large electrode pads (10 × 15 cm) (Caremax, Australia) placed over the anterior surface of the thigh. Each session of NMES will involve both low- (4 Hz) and high-frequency (50 Hz) stimulation with a pulse width of 300 µs. Participants will have control of the stimulation current throughout the session with guidance from the supervising investigator. Participants will be supine or sitting up/semi-reclined in a bed or dialysis chair with a triangular foam wedge (or similar alternative) placed under the participant’s knees to increase the joint angle (~70° from full extension) to maximise the contractile response and enhance comfort. A strap across the ankles will keep the participant’s legs securely fastened in place (Fig. 2). NMES will normally occur during the first hour of dialysis, when the participant is feeling less fatigued. For individuals that dialyse at home, they will be able to perform the intervention at a time that is convenient for them.

The NMES session protocol was developed following systematic pilot investigations applying different acute NMES permutations with HD participants and following our previous NMES intervention study [25]. Low-frequency stimulation will be delivered at both the start (warm-up, 5 min) and end (cool-down, 5 min) of the dialysis session to both legs simultaneously. Participants will be encouraged to increase the current as follows: first session to a discomfort of 4/10 (between ‘mild’ and ‘moderate discomfort’); subsequent sessions, initially 90% of the highest warm-up current from all previous sessions, and then reach or exceed the highest previous warm-up current.

The high-frequency stimulation (frequency: 50 Hz, pulse width: 300 µs) will involve contractions of 6 s in duration (with a 1 s ramp increase, 4 s plateau, and 1 s ramp decrease in current) with 11 s rest between contractions, alternating between sets on each leg (i.e. left, right, left, etc.). The high-frequency NMES will be progressed as follows:

1. Increasing session duration: the duration of high-frequency stimulation will be increased up to 30 min by progressively increasing the number of sets and repetitions performed.
2. Increasing stimulation intensity: the first session will establish a starting current based on the participant’s tolerance and discomfort. Thereafter, participants will be encouraged to gradually increase the current during each session and over subsequent sessions, potentially reaching or exceeding their previously achieved maximum.

The duration, maximum frequency, discomfort (0–10), and rating of perceived exertion (RPE; 0–10) will be recorded at the end of the session. Exercise intensity will also be assessed by measuring blood lactate at the end of a session once per week (excluding home dialysis patients due to logistical constraints of sample collection in the home setting). Standard care was selected as the comparator because it reflects current routine management for patients receiving dialysis.

### Criteria for discontinuing or modifying allocated intervention/comparator {15b}

Participants may withdraw from complying with the allocated study treatment arm and/or providing data to the study, at any time, for any reason, without affecting their usual care. Should a participant wish to withdraw, efforts will be made to continue to obtain follow-up data, with their permission. Participants do not have to provide a reason for withdrawal; however, if they do provide a reason for leaving the study, this will be recorded.

The clinical team and/or investigators may discontinue a participant from the study at any time if considered necessary, e.g. based on symptoms, transplantation, change in dialysis modality, etc.

### Strategies to improve adherence to intervention/comparator {15c}

Dedicated research staff will be based at each dialysis unit to support participants in using the NMES device and to provide encouragement throughout each session. If a participant experiences difficulty on a given day, they will be advised to lower the stimulation intensity rather than skip the session entirely, in order to maintain adherence to the intervention. Each NMES session will be logged in a case report form (CRF) to allow progress to be closely monitored.

### Concomitant care permitted or prohibited during the trial {15d}

All other aspects of dialysis care will remain the same during participation in the STIM-HD study. Individuals will be permitted to take part in other interventions as long as there are no conflicting therapies or outcomes.

### Ancillary and post-trial care {34}

No specific post-test care will be provided as part of this study. Given NMES devices are commercially available, participants may choose to purchase a personal device at the end of the study, irrespective of group allocation.

### Outcomes {16}

**Fig. 3:**
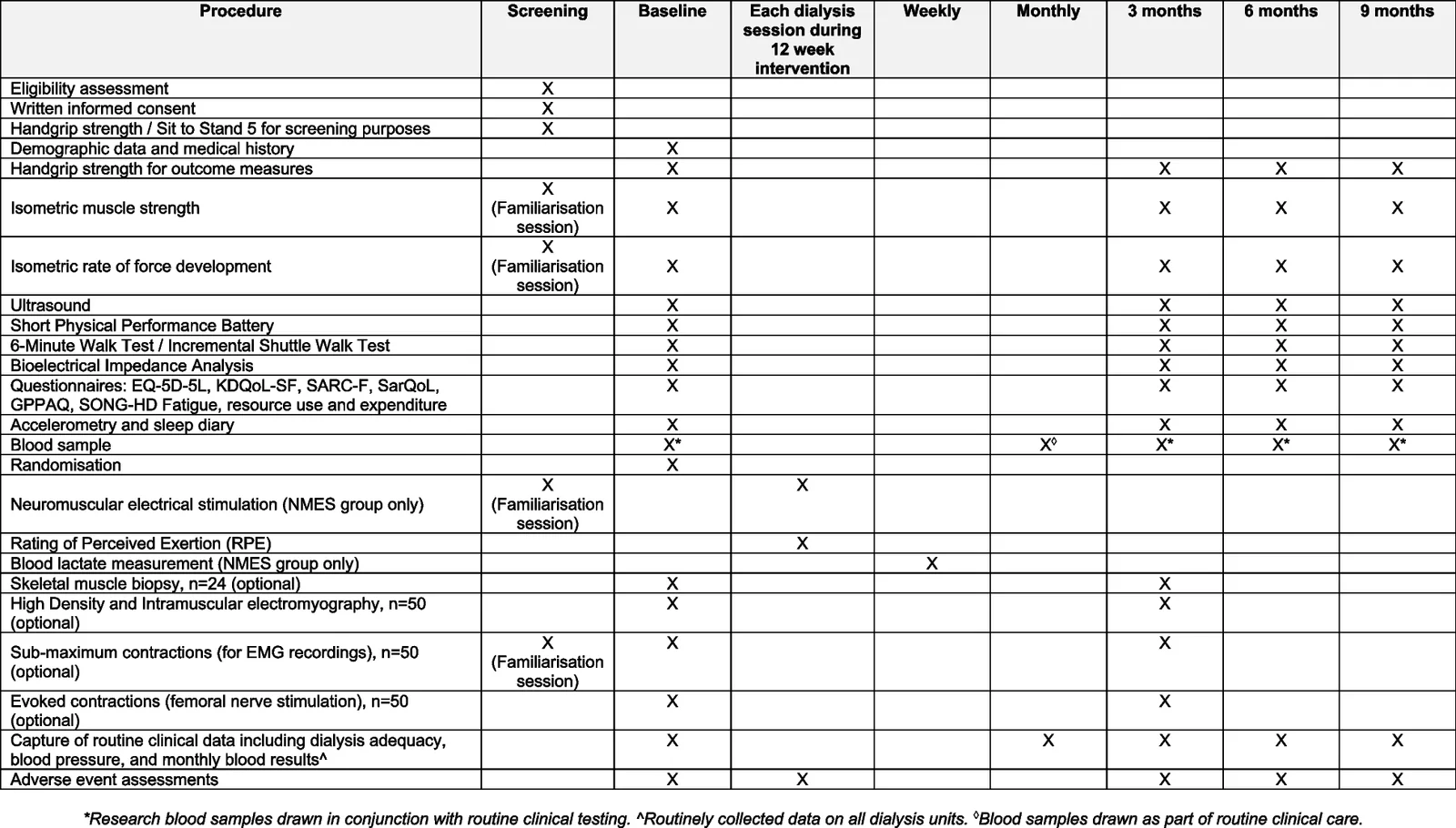
Schedule of enrolment, randomisation, assessments, and follow-up

The primary outcome measure will be assessed at baseline and 3 months post randomisation. Secondary outcome measures will be assessed at baseline, 3 months, 6 months, and 9 months post randomisation. Mechanistic outcome measures will be measured at baseline and 3 months only (Fig. 3).

#### Primary outcome

The primary outcome of this study is change in knee extension muscle strength from baseline to 3 months measured using isometric dynamometry (Newton-metres (Nm)).

Although confirmed sarcopenia is defined as deficits in both muscle strength and muscle mass, with severity graded by physical function levels, the single primary outcome measure will be muscle strength for several reasons. Firstly, the NICE guidance (IPG677) recommends considering the use of electrical stimulation to increase muscle strength. Secondly, the European Working Group on Sarcopenia in Older People (EWGSOP2) guidelines identify reduced muscle strength as the primary manifestation of sarcopenia and the symptom of greatest importance. These guidelines recommend that interventions to arrest or reverse its progression should be introduced at the ‘probable’ stage that is defined by low strength alone [26]. Thirdly, our PPIE group indicated that the most important clinical outcome to people with kidney disease is functional ability, which is determined by muscle strength and physical endurance, rather than mass.

#### Secondary outcomes

The secondary outcome measures (Table 2) will further explore effects upon muscle mass and architecture; evaluate physical function, body composition, and physical activity levels; explore quality of life and NHS resource use; assess safety of the intervention and impact on clinical parameters; and determine adherence to the intervention. These secondary outcome measures also include mechanistic assessments (Table 3) that will be investigated in work package 4 (optional mechanistic outcomes study).

**Table 2:** Secondary outcome measures and method of assessment for work package 2 (randomised controlled trial)

Domain	Measure	Metric	Method of aggregation	Timepoint
Muscle strength	Knee extension muscle strength (change from baseline)	On isometric dynamometry (Nm)	Between-group adjusted mean difference (95% CI)	6 months and 9 months (with baseline measure for comparison)
Muscle mass	Ultrasound of rectus femoris and vastus lateralis (change from baseline)	Muscle cross-sectional area (cm^2^) and thickness (mm)	For rectus femoris: between-group adjusted mean difference (95% CI) For vastus lateralis: summary statistics: mean (standard deviation)	3 months, 6 months, and 9 months (with baseline measure for comparison)
Muscle architecture	Ultrasound for pennation angle and fascicle length of both rectus femoris and vastus lateralis (change from baseline)	Pennation angle (degrees) and fascicle length (mm)	Summary statistics: mean (standard deviation)	3 months (with baseline measure for comparison)
Muscle quality	Ultrasound for echo intensity	Echo intensity (mean grayscale value; 0–255 arbitrary units)	Summary statistics: mean (standard deviation)	3 months (with baseline measure for comparison
Physical function	1. Short Physical Performance Battery (SPPB) (change from baseline) 2. 6-min walk test (6MWT) (change from baseline) 3. Handgrip strength (change from baseline)	1. Short Physical Performance Battery (SPPB) total score (0–12 points) 2. Total distance walked (metres) 3. Maximum grip strength force (kg)	Between-group adjusted mean difference (95% CI)	3 months, 6 months, and 9 months (with baseline measure for comparison)
Body composition	Bioelectrical impedance analysis (BIA) (change from baseline)	Body fat %, body fat mass (kg), lean mass (kg)	Summary statistics: mean (standard deviation)	3 months, 6 months, and 9 months (with baseline measure for comparison)
Physical activity levels	1. GENEActiv accelerometery device (change from baseline) 2. GP Physical Activity Questionnaire (GP PAQ)	1. a) Average acceleration (proxy for volume physical activity (mg)) b) Time spent inactive (sedentary time) (minutes/day) c) Time spent in light intensity physical activity (minutes/day) d) Time spent in moderate-vigorous physical activity (MVPA) bouts of ≥1 min during waking hours (minutes/day) e) Total sleep or actual sleep duration gained throughout the sleep window, excluding any awake time (hours/day) 2. Categorical index categories (e.g. active, inactive, moderately inactive, moderately active)	1. Between-group adjusted mean difference (95% CI) 2. Proportion (%) of participants in each PAQ category at each timepoint	3 months, 6 months, and 9 months (with baseline measure for comparison)
Quality of life and social functioning	Change from baseline in Kidney Disease Quality of Life - Short Form (KDQoL-SF), EuroQoL EQ-5D-5L, Standardised Outcomes in Nephrology - Haemodialysis (SONG-HD), Strength, Ambulation, Rising from a chair, Stair climbing and history of Falling (SARC-F), Sarcopenia related Quality of Life (SarQoL)	Questionnaire-specific summary or utility index scores (i.e. 0–100 score for KDQoL, index values/visual analogue scale (VAS) score for EQ-5D-5L, 0–9 for SONG-HD, 0–10 for SARC-F, 0–100 for SarQoL)	Between-group adjusted mean difference (95% CI)	3 months, 6 months, and 9 months (with baseline measure for comparison)
NHS resource use	Resource usage questionnaire	Self-reported frequency of healthcare interactions (e.g. GP visits, emergency admissions) or estimated total direct cost (£)	Cumulative total or mean cost/frequency per participant over the study duration	3 months, 6 months, and 9 months (with baseline measure for comparison)
Cost-effectiveness	Combined assessment of: 1. Resource usage questionnaire (costs) 2. EQ-5D-5L/KDQoL-SF (health utilities)	Incremental Cost-Effectiveness Ratio (ICER) expressed as cost (£) per quality-adjusted life year (QALY) gained	Incremental Cost-Effectiveness Ratio (ICER) and Incremental Net Benefit calculated from the mean differences in costs and QALYs between arms	Over the 9-month follow-up period
Measures of process and tolerability	NMES (neuromuscular electrical stimulation) session log	Total number of NMES sessions completed and average sets/reps completed per session	Median (and interquartile range) of NMES sessions, sets, and repetitions achieved across participants	Intervention period
Measures of safety	Adverse event (AE) reporting logs	1. Count, type, and severity grading (e.g. mild/moderate/severe) of AEs deemed causally related to study intervention 2. Count, type, and severity grading of AEs deemed causally related to study procedures	Total number of events and the proportion (%) of participants experiencing at least one related AE per group	Over the 9-month follow-up period
Measures of safety	Serious adverse event (SAE) reporting logs	Count, type, and severity grading of SAEs	Total number of events and the proportion (%) of participants experiencing at least one SAE per group	Over the 9-month follow-up period

**Table 3:** Secondary outcome measures and method of assessment for work package 4 (mechanistic outcomes study)

Domain	Measure	Metric	Method of aggregation	Timepoint
Skeletal muscle fibre cross-sectional area/myofibrillar area	Vastus lateralis muscle biopsy, immunofluorescence microscopy	Mean muscle fibre cross-sectional area per participant (µm^2^)	Between-group mean difference	Baseline and 3 months
Skeletal muscle fibre type composition	Vastus lateralis muscle biopsy, immunofluorescence	Proportion of fibres classified as type I, type IIa, and type IIx, expressed as a percentage of fibres assessed	Between-group mean difference	Baseline and 3 months
Skeletal muscle ultrastructure	Vastus lateralis muscle biopsy, transmission electron microscopy	Mean sarcomere length per participant (µm) Mean myofilament spacing per participant (nm)	Between-group mean difference	Baseline and 3 months
Skeletal muscle mitochondrial content	Vastus lateralis muscle biopsy; citrate synthase activity assay	Citrate synthase activity normalised to total protein (mU/mg protein)	Between-group mean difference	Baseline and 3 months
Skeletal muscle mitochondrial content	Vastus lateralis muscle biopsy; quantitative PCR	Relative mitochondrial DNA copy number, normalised to nuclear DNA	Between-group mean difference	Baseline and 3 months
Skeletal muscle mitochondrial content	Vastus lateralis muscle biopsy; western blotting	Porin protein abundance, normalised to loading control or total protein (arbitrary units)	Between-group mean difference	Baseline and 3 months
Skeletal muscle mitochondrial respiratory complex abundance	Vastus lateralis muscle biopsy; western blotting	Relative abundance of representative proteins from mitochondrial respiratory chain complexes I–V, normalised to loading control or total protein (arbitrary units)	Between-group mean difference	Baseline and 3 months
Skeletal muscle mitochondrial biogenesis	Vastus lateralis muscle biopsy; quantitative reverse-transcription PCR	Relative expression of prespecified mitochondrial biogenesis genes, expressed as 2^−ΔΔCt^	Between-group mean difference	Baseline and 3 months
Skeletal muscle mitophagy	Vastus lateralis muscle biopsy; quantitative reverse-transcription PCR	Relative expression of prespecified mitophagy genes expressed as 2^−ΔΔCt^	Between-group mean difference	Baseline and 3 months
Skeletal muscle mitochondrial respiratory function	Permeabilised vastus lateralis muscle fibres; high-resolution respirometry using the OROBOROS Oxygraph-2k	Oxygen flux for prespecified mitochondrial respiratory states, normalised to tissue mass [pmol O_2_·s^−1^·mg^−1^]	Between-group mean difference	Baseline and 3 months
Mitochondrial ultrastructure	Vastus lateralis muscle biopsy; transmission electron microscopy	Mitochondrial area (µm^2^)	Between-group mean difference	Baseline and 3 months
Mitochondrial ultrastructure	Vastus lateralis muscle biopsy; transmission electron microscopy	Mitochondrial number density (mitochondria/µm^2^)	Between-group mean difference	Baseline and 3 months
Mitochondrial ultrastructure	Vastus lateralis muscle biopsy; transmission electron microscopy	Mitochondrial volume density (%)	Between-group mean difference	Baseline and 3 months
Mitochondrial ultrastructure	Vastus lateralis muscle biopsy; transmission electron microscopy	Cristae density or cristae surface density	Between-group mean difference	Baseline and 3 months
Mitochondrial ultrastructure	Vastus lateralis muscle biopsy; transmission electron microscopy	Mitochondrial circularity/aspect ratio	Between-group mean difference	Baseline and 3 months
Skeletal muscle proteome	Vastus lateralis muscle biopsy; untargeted LC-MS/MS proteomics	Relative abundance of identified proteins (log_2_-transformed normalised intensities)	Between-group mean difference. Multiple testing controlled using the Benjamini-Hochberg false discovery rate (FDR)	Baseline and 3 months
Skeletal muscle transcriptome	Vastus lateralis muscle biopsy; bulk RNA sequencing	Normalised gene-expression counts and log_2_ fold-change in expression of individual genes	Between-group mean difference. Multiple testing controlled using the Benjamini-Hochberg false discovery rate (FDR)	Baseline and 3 months
Plasma transcriptome	Plasma, untargeted LC-MS/MS proteomics	Relative abundance of identified proteins (log_2_-transformed normalised intensities)	Between-group mean difference. Multiple testing controlled using the Benjamini-Hochberg false discovery rate (FDR)	Baseline and 3 months
Neuromuscular junction instability	Intramuscular electromyography (iEMG) of vastus lateralis	Motor unit jiggle, motor unit jitter	Between-group mean difference	Baseline and 3 months
Neuromuscular junction instability	Plasma, ELISA	Circulating concentrations of C-terminal agrin fragment	Between-group mean difference	Baseline and 3 months
Motor unit firing frequency and conduction velocity	High-density surface EMG (HD-sEMG) of vastus lateralis and vastus medialis	Motor unit firing frequency, motor unit conduction velocity	Between-group mean difference	Baseline and 3 months
Quadriceps contractile properties	Percutaneous femoral nerve stimulation	Peak twitch torque	Between-group mean difference	Baseline and 3 months

### Harms {17}

Participant safety will be monitored for the duration of the 9-month follow-up period. It is expected that participants will experience a significant number of underlying health conditions and consequently an increased number of expected hospital admissions [27]. In addition, as this is an interventional study, expected events may occur related to the NMES intervention.

Common, non-life-threatening adverse events (AEs) (such as constipation, diarrhoea, colds, and headache) will not be reported in the study. Only AEs causally related to the study procedures or NMES intervention will be recorded. The causal relationship of AEs to the procedures or intervention will be assessed and signed off by a medically qualified individual delegated this responsibility on the study Delegation of Activities Log.

Serious adverse events (SAEs) will be collected and are included in the secondary outcomes (Table 2). SAE data will be presented at regular Data Safety Monitoring Committee (DSMC) meetings for review of clinical relevance. Findings from these reviews will inform the Trial Steering Committee (TSC) and sponsor regarding the appropriateness of study continuation, as well as the potential need for additional data and/or analyses.

AEs will be assessed using both systematic and non-systematic approaches. A structured AE checklist will be completed by researchers at scheduled visits and NMES sessions, while additional AEs will be documented through spontaneous participant reports.

Expedited reporting to the sponsor will only occur when SAEs are clearly related to the study procedures or intervention and are of a serious nature. These SAEs include:

- Musculoskeletal injury as a direct result of the outcome assessments, skeletal muscle biopsy procedure, EMG, or the intervention (NMES)
- Anything else in the investigator’s opinion that is related, serious, and unexpected

Deaths will be recorded as part of the study outcomes and therefore will not be subject to expedited reporting.

All SAEs will be tabulated and summarised by treatment group according to system organ class, using Medical Dictionary for Regulatory Activities (MedDRA) system organ class categories; full MedDRA coding will not be performed. No formal statistical testing will be performed. All events will be summarised by seriousness, expectedness, and relatedness.

### Participant timeline {18}

Overview of the participant timeline and schedule of events is summarised in Fig. 3, which includes the schedule of enrolment, randomisation, intervention period, outcome assessments, and follow-up.

### Sample size {19}

The recruitment target is 228 participants: 114 to the intervention arm and 114 to the standard care arm. This recruitment target consists of both the sample size required to achieve study power and considerations for participant drop-out.

The study is powered to detect a 21.7 Nm (SD 47.5 Nm) difference (standardised effect size of 0.5 [moderate]) in change in muscle strength between groups at 3 months with 90% power at the 5% significance level. This will also account for an expected 10% total attrition rate, based on our previous data [15].

No minimal clinically important difference (MCID) has been reported for muscle strength as determined by isometric dynamometry in the CKD population. However, our data have shown that a 10 Nm increase in muscle strength was associated with a reduction in 3-year mortality of 5% [28]. The data we have used in our sample size calculation come from the improvement in strength that has been reported by three previous studies of NMES in haemodialysis patients [20, 21, 23, 24], i.e. a mean strength improvement of 21.7 Nm and a mean standard deviation of 47.5 Nm.

Recruitment will be reviewed by the independent TSC and DSMC to ensure the study remains adequately powered with respect to participant retention and permanent treatment arm crossover. Although every effort will be made to minimise crossovers from both intervention arms, the numbers, direction, and reasons for participants moving between arms will be recorded and reported in line with CONSORT (Consolidated Standards of Reporting Trials) guidance.

### Recruitment {20}

The inclusion criteria for the STIM-HD study are broad, allowing most adults receiving haemodialysis to participate in the main RCT. Initially, patients at participating dialysis units will undergo pre-screening to identify adults who have been receiving HD (either at home or in-centre) for at least 3 months and who do not meet any exclusion criteria. Exclusions include contraindications to NMES therapy, conditions known to cause muscle wasting (such as diagnosed neuromuscular diseases or active malignancy), scheduled living donor kidney transplantation, a life expectancy of less than 6 months, or current participation in an interventional trial with conflicting therapies or outcomes.

Potential participants will be identified by a member of the clinical team based upon the exclusion and inclusion criteria. They will be recruited via the following routes:

- During or before a routine outpatient clinical appointment
- During a routine dialysis session or ward round on the dialysis unit

Patients interested in the study will be provided with a participant information sheet and given a minimum of 48 h (i.e. 2 days between routine HD sessions) before they are revisited for re-discussion. This re-discussion will either result in informed consent (with progression to screening), study participation declined, or study discussions paused (e.g. the participant may require more time to consider their decision).

Participants will be recruited from approximately five dialysis units from up to five NHS Trusts. In the event of recruitment challenges, there is also the option to expand enrolment to other dialysis units within these networks. The study commenced in May 2025 and will close to recruitment in December 2026.

## Assignment of interventions: randomisation

### Sequence generation: who will generate the sequence {21a}

The allocation sequence will be generated by the secure web-based randomisation system [29].

### Sequence generation: type of randomisation {21b}

After providing written informed consent and successfully completing the sarcopenia screening, participants will be randomised in a 1:1 manner to receive either the NMES intervention or continue with standard care. To ensure external validity of the results, randomisation will be completed using minimisation factors of age (<60 years and ≥60 years), time on dialysis (<1 year and ≥1 year), and site (dialysis unit). Only designated members of the study team will be responsible for performing randomisation. Randomisation results will be revealed to the designated researcher after baseline measurements have been collected.

### Allocation concealment mechanism {22b}

Randomisation will be completed via a web-based system [29] to maintain allocation concealment.

### Implementation {23}

All study team members who have completed the required training will be authorised to obtain informed consent, confirm eligibility requirements, and perform baseline outcome assessments. To maintain allocation concealment, only designated members of the study team will be responsible for generating the allocation sequence and performing randomisation. Treatment allocation will be disclosed solely to individuals administering the NMES intervention in order to maintain blinding of outcome assessors.

## Assignment of interventions: blinding

### Who will be blinded {24a}

Outcome assessors and data and sample analysts will remain blinded throughout the study. Study team members administering the intervention will not be blinded and therefore will not perform any further outcome measures after the baseline assessments.

### How will blinding be achieved {24b}

To further maintain allocation concealment during the study, the intervention will be delivered with the curtains drawn around participant’s beds or dialysis chairs, when possible. The curtains will also be drawn around the bed of those individuals assigned to standard care for the same duration. Due to the nature of the intervention, it is not possible to blind the participants to the treatment allocation.

### Procedure for unblinding if needed {24c}

Unblinding of assessors will only occur in exceptional circumstances where knowledge of group allocation is deemed essential for the participant’s safety or for the clinical management of a serious adverse event. Any decision to unblind will be made by the Chief Investigator and will be documented, including the reason for unblinding, the personnel involved, and the time and date. The assessor who is unblinded will no longer participate in blinded data collection for that participant.

## Data collection and management

### Plans for assessment and collection of outcomes {25a}

Data collection will be conducted by the research delivery teams at participating sites during routine haemodialysis appointments. This is applicable for both in-centre and home-based haemodialysis patients. Participants receiving home haemodialysis will visit their local unit in order to take part in outcome assessments and, if allocated to the NMES intervention, to receive training on its use. Clinical information and results will be collected from routine clinical testing and medical records. Data entry will be completed by the researchers at participating sites.

Standard operating procedures (SOPs) have been developed for each outcome measure, and all research staff have received comprehensive training to ensure consistency and data integrity. For the muscle strength assessment, staff were required to complete the full protocol on five naïve individuals on three distinct occasions, with the resulting data reviewed for both quality and reproducibility. For the strength measurements, following a brief warm-up, participants will perform three maximum voluntary isometric contractions on a custom-built, low-noise, rigid seated isometric knee extension dynamometer, with the knee and hip set at 115° and 126° respectively. Extraneous movement will be minimised using adjustable straps across the pelvis and shoulders. A reinforced 35-mm canvas ankle strap will be positioned just superior to the medial malleolus of the ankle (approximately 15% of tibial length) and connected in series to a calibrated S-beam strain gauge (Force Logic, Swallowfield, UK) orientated posterior and perpendicular to the tibia. The analogue force signal will be amplified (×370), sampled continuously at 2000 Hz via an external A/D convertor (Micro 1401, CED, Cambridge, UK), and recorded in Spike 2 software (CED Ltd, Cambridge, UK). Following offline filtering and gravity correction, peak instantaneous force will be multiplied by lever length (the distance from the knee joint space to the centre of the ankle strap) to calculate maximum voluntary torque (isometric strength). For the ultrasound assessments, assessors will capture three separate images per measurement. Only data where image-derived measurements fall within 10% of each other will be considered valid and included in the analysis.

For outcome measures where a learning effect is anticipated, specifically isometric rate of force development and neuromuscular mechanistic measures requiring steady submaximal force production, participants will undergo a familiarisation session prior to formal data collection to ensure reliable and representative performance.

### Plans to promote participant retention and complete follow-up {25b}

Study participation on the allocated treatment arm is 3 months with additional follow-ups at 6 and 9 months. Outcome assessments performed at all time points can be split over multiple visits if this is more convenient for the participant.

Each dialysis unit will have two or more dedicated research staff to support participant engagement and promote ongoing participation. Participants in the NMES intervention group will be visited at each dialysis session to support consistent use of the device; support will be provided as required. Participants in the standard care group will also be visited each dialysis session; curtains will be drawn around their bed, when possible, to maintain allocation concealment and this continuous interaction with the study team will encourage maintained participation in the study. To maximise retention, research staff will build rapport with participants and maintain regular, supportive communication throughout the study.

### Data management {26}

Study data will be collected and managed using a web-based Remote Data Capture system [30, 31], provided by Leicester Clinical Trials Unit. The database will be located on a University of Leicester secure server. Unique study identifiers will be assigned to each participant. The database includes pre-defined reference ranges for each outcome measure, which will automatically flag any data points which fall outside these thresholds. This will help prevent incorrect data entry. All paper CRFs will be kept in locked cabinets at each participating site until the end of the study retention period (6 years after the study ends). Ultrasound scan images and data generated during the strength and EMG tests will be stored externally to the Research Electronic Data Capture (REDCap) database and digitally labelled with unique study identifiers. The study will be conducted in compliance with the approved protocol and will adhere to the principles outlined in the UK Policy Framework for Health and Social Care Research (2017), ICH-GCP guidelines, the sponsor’s (and any other relevant) SOPs, and other relevant regulatory requirements.

### Confidentiality {33}

Each participant will be assigned a unique identification number upon randomisation.

Participants’ personal data included in study-related databases shall be treated in confidence and in compliance with ICH-GCP, the UK Policy Framework for Health and Social Care, and the EU General Data Protection Regulation (GDPR). When processing or archiving personal data, the sponsor or its representative shall take all appropriate measures to safeguard and prevent access to this data by any unauthorised third party.

The REDCap database will be password-protected and only authorised members of the study team will have access.

## Statistical methods

### Statistical methods for primary and secondary outcomes {27a}

A statistical analysis plan (SAP) will be drafted and agreed with appropriate oversight prior to data analysis. Any changes to the original SAP will be detailed along with the reason(s) for changes in subsequent SAPs.

Primary outcome analysis: This will compare the change in muscle strength from baseline to 3 months (i.e. at the end of the intervention period) between randomised groups. Linear regression with change in muscular strength as the dependent variable, adjusted for baseline value and the minimisation factors (age, time on dialysis, site (dialysis unit)), will be used to compare treatment groups; the adjusted mean difference between the treatment arms will be presented with a 95% confidence interval.

Secondary outcome analysis: Secondary outcomes will be analysed using appropriate statistical models, with intercurrent events handled in a manner consistent with the primary outcome analysis. All represent continuous data and therefore linear regression will be used. All analyses will be adjusted for baseline value and the minimisation factors. The number of deaths and cardiovascular events expected is relatively low; therefore, formal time to event analyses will not be conducted. Kaplan–Meier will be presented to describe the relationship between these events and treatment.

### Who will be included in each analysis {27b}

The primary outcome will be analysed in the intention-to-treat (ITT) population, comparing participants randomised to the NMES intervention with those randomised to standard care. Participants who receive a kidney transplant prior to the 3-month assessment will be excluded from the analysis, consistent with a principal stratum strategy within the estimand framework. Participants who die before the 3-month timepoint and therefore do not contribute outcome data will also be excluded, corresponding to a while-alive strategy.

Secondary outcomes will be analysed using a complete-case approach, with intercurrent events handled in a manner consistent with the primary outcome analysis.

### How missing data will be handled in the analysis {27c}

By design there will be no missing data for the minimisation factors. If the primary outcome is missing for less than 5% of the study participants at baseline and 3 months, a complete-case analysis will be conducted. If there is more than 5% missing outcome data, multiple imputation (MI) will be used. The imputation will be carried out using the command MI in Stata. MI replaces missing values with multiple sets of simulated values to complete the data, performs standard analysis on each completed dataset, and adjusts the obtained parameter estimates for missing-data uncertainty using Rubin’s rules to combine estimates. If imputation is required, sensitivity analyses will be conducted to assess the robustness of results to the missing-at-random (MAR) assumption underlying the imputation of missing data. All secondary outcomes will be analysed using a complete-case approach.

### Methods for additional analyses (e.g. subgroup analyses) {27d}

For the primary outcome, a priori subgroup analysis will include gender, age (<60 years and ≥60 years), time on dialysis (<1 year and ≥1 year), physical activity levels (i.e. sedentary versus active), and the severity of sarcopenia at baseline (i.e. probable vs severe). Descriptive analyses within subgroups will be performed to show the difference in mean change from baseline between treatment groups. Interaction terms will be used to assess the between subgroup difference in treatment effectiveness.

A health economic evaluation will be conducted to assess the cost-effectiveness of neuromuscular electrical stimulation (NMES) programmes in patients receiving HD. Healthcare-related resource use will be collected and combined with unit costs using standard validated external tools [32–34]. Quality-adjusted life years (QALYs) will be derived from EQ-5D-5L data. Cost and QALY data will be used to calculate the incremental cost per QALY gained. Study results will be extrapolated to explore the potential costs and effect over the longer term.

### Interim analyses {28b}

No formal stopping rules or interim analyses have been pre-defined or planned.

### Protocol and statistical analysis plan {5}

The protocol will be publicly accessible upon publication. The statistical analysis plan will be submitted as an update prior to data lock.

### Plans to give access to the full protocol, participant-level data, and statistical code {31c}

Applications to access anonymised patient-level data can be made to the Trial Management Group (TMG). The full protocol will be available as a supplement upon publication of the primary results paper.

## Oversight and monitoring

### Composition of the coordinating centre and trial steering committee {3d}

The study is sponsored by the University of Leicester and coordinated by the central research team, comprising research assistants and researchers who are specifically responsible for participant recruitment, intervention implementation, and data collection across participating sites. The coordinating centre will be overseen by the TMG, led by the co-lead applicants and senior trial manager. The TMG will meet regularly to monitor overall study progress and management, including site set-up, recruitment, completion, safety, protocol implementation and review, and emerging issues. The TMG includes a PPIE co-applicant who contributed to the development of the research question and study design. This co-applicant will provide the patient perspective to the TMG and chair regular PPIE meetings to gather patients’ experiences of both the study and the intervention.

An independent TSC will provide overall supervision on behalf of the study’s sponsor and funder. The TSC will monitor study progress, ensure adherence to the protocol, and assess the scientific quality of the study. It will provide expert, independent advice on ethical and operational matters, while safeguarding the rights and well-being of participants. The TSC will meet at least annually or as required throughout the study and will comprise an independent chair, an expert statistician, a health economist, a clinician with relevant expertise, and a lay representative.

### Composition of the data monitoring committee, its role and reporting structure {28a}

The independent DSMC will provide impartial oversight of the study’s safety data and emerging efficacy findings and will operate independently of the sponsor and study team. The DSMC will review unblinded interim data and make recommendations to the TSC and sponsor regarding the continuation, modification, or early termination of the study, based on participant safety, data integrity, and ethical considerations. The DSMC will meet at least annually or as required throughout the study and will comprise an independent chair, expert statistician, and a clinician with relevant expertise.

### Frequency and plans for auditing trial conduct {29}

The sponsor operates a risk-based monitoring and audit programme to which this study will be subject. Leicester Clinical Trials Unit operates a Quality Management System which will apply to this study, with quality checks and quality assurance audits performed as required.

### Protocol amendments {31}

All protocol amendments require approval from the sponsor, funder, Research Ethics Committee (REC), Health Research Authority (HRA), and site Research and Development/Innovation (R&D/I) departments prior to implementation. Where appropriate, study participants will be notified of protocol amendments, and the requirement for this communication will be determined by the sponsor and REC.

### Dissemination policy {8}

The study results will be disseminated widely to the nephrology and wider clinical community, as well as patients living with kidney disease, through national and international conferences, peer-reviewed journal publications, PPIE newsletters and forums, and social media.

## Discussion

Sarcopenia is prevalent in the haemodialysis population and is clinically significant due to the negative impact on quality of life, morbidity, and mortality. Currently, no established pharmacological therapies exist for sarcopenia. Exercise remains the only intervention with demonstrated efficacy, enhancing muscle mass and strength in people with sarcopenia receiving haemodialysis [9, 10]. Recent UK Kidney Association (UKKA) guidelines, endorsed by the NICE, recommend structured, dialysis unit-based exercise programmes as part of routine care, reflecting the recognised role of physical activity in improving functional outcomes and quality of life. However, despite the well-established cardiovascular and musculoskeletal benefits of traditional exercise programmes, up to 74% of individuals receiving haemodialysis are unable or unwilling to participate [12, 13].

This protocol outlines a rigorous study investigating NMES in sarcopenic haemodialysis patients, with muscle strength as the primary outcome measure. Beyond functional and quality of life outcomes, the study will comprehensively examine NMES-induced neuromuscular adaptations, including motor unit activity, neuromuscular junction instability, mitochondrial health, and transcriptional and proteomic changes. These mechanistic analyses will enhance understanding of both the presence and absence of NMES effects. If NMES proves effective, these insights will guide adjunct strategies, such as targeted nutritional support, and provide valuable information on the underlying contributors to sarcopenia in this population, an area where current understanding remains limited.

A key consideration in multi-centre RCT planning is the preservation of assessor blinding. To minimise bias, outcome assessors will remain blinded to group allocation, supported by standardised procedures such as separate staff for intervention delivery and outcome assessments, uniform scheduling processes, and cross-site training. Dedicated research staff will visit participants in both study arms to maintain intervention parity and reduce the risk of accidental unblinding. Given the multi-centre design, inter-operator variability presents a potential source of error. This risk has been minimised through the use of standardised protocols, comprehensive training, and by ensuring that each participant’s outcome measures are performed by a single assessor.

The strength of our design lies in its multi-modal mechanistic approach, robust outcome assessor blinding, and carefully standardised procedures. While the haemodialysis population with sarcopenia is specific, which may limit generalisability, and participant characteristics such as age, comorbidities, and baseline physical activity could influence NMES responses, the above-mentioned strategies are designed to optimise adherence, minimise bias, and maintain data quality. Should the study yield positive results, we will work to support the integration of NMES into clinical practice.

## Trial status

The protocol described within this manuscript is according to the most recent study protocol (version 4.0, 16/01/2026). Details of previous versions of the protocol can be found in Table 4. The STIM-HD grant period began on 01/09/2024. Study activities commenced on 01/05/2025 following receipt of governance approvals and the first participant was enrolled on 30/06/2025. The recruitment period is scheduled to end on 31/12/2026. The overall study end date is 29/02/2028. The current protocol version is 4.0, 16 January 2026.

**Table 4:** Study protocol revision chronology

Version and date	Reason for amendment
V1.0 17/10/2024	Initial version submitted to the Research Ethics Committee (REC) and Health Research Authority (HRA)
V2.0 31/03/2025	Clarifications and corrections, updates to outcome measures and timepoints, updates to the randomisation process, administrative corrections to the eligibility criteria
V3.0 23/07/2025	Implementation of a two-tiered screening approach comprising handgrip strength and sit to stand 5 test. Administrative corrections to study flow chart and schedule of procedures. Minor refinements to exclusion criteria. Clarification added specifying that participants should commence the intervention within 2 weeks of randomisation. Work package 4—optional mechanistic study: additional biopsy for the NMES group removed. Clarification added that participants may choose to take part in all, some, or none of the optional assessments. Familiarisation session for femoral nerve stimulation removed. Additional detail added regarding participant positioning during NMES sessions
V4.0 16/01/2026	Administrative updates; addition of protocol contributors. Sit to stand 60 test (muscle endurance) removed from secondary endpoints/outcomes. Reference to specific NHS Trusts removed. Metallic hip or knee joint removed from exclusion criteria. Sit to stand 60 removed from schedule of procedures. Timeline for randomisation changed from after baseline measurements have been collected to after providing written informed consent and successfully completing sarcopenia screening. Clarification added that randomisation results will be revealed to the designated researcher after baseline measurements have been collected Clarification added that participants can position their legs over a foam wedge or similar alternative. Clarification added that home dialysis patients will be excluded from blood lactate measurements due to logistical constraints. Participants with metal objects in/around the hip joint will no longer be excluded from the femoral nerve stimulation assessment

## Supplementary Information

Supplementary Material 1.

## Data Availability

Applications to access anonymised patient-level data can be made to the Trial Management Group (TMG). The full protocol will be available as a supplement upon publication of the primary results paper. RNA sequencing data will be deposited with the NCBI Gene Expression Omnibus.

## Abbreviations

AE: Adverse event
BIA: Bioelectrical impedance analysis
CKD: Chronic kidney disease
CONSORT: Consolidated Standards of Reporting Trials
CRF: Case report from
CTU: Clinical Trials Unit
DSMC: Data Safety Monitoring Committee
EMG: Electromyography
EQ-5D-5L: EuroQol EQ-5D-5L—Quality of Life questionnaire
ESKD: End-stage kidney disease
EWGSOP2: European Working Group on Sarcopenia in Older People
ICH-GCP: International Council for Harmonisation—Good Clinical Practice
GP PAQ: General Practice Physical Activity Questionnaire
GDPR: General Data Protection Regulation
HD: Haemodialysis
HRA: Health Research Authority
ICH-GCP: International Council for Harmonisation of Technical Requirements for Pharmaceuticals for Human Use—Good Clinical Practice
iEMG: Intramuscular electromyography
ISRCTN: International Standard Randomised Controlled Trials Number
ITT: Intention-to-treat
KDQoL: Kidney Disease Quality of Life instrument
KDQoL-SF: Kidney Disease Quality of Life—Short Form
MAR: Missing-at-random
MCID: Minimal clinically important difference
MI: Multiple imputation
miRNA: MicroRNA
MVPA: Moderate-to-vigorous physical activity
NHS: National Health Service
NICE: National Institute for Health and Care Excellence
NIHR: National Institute for Health and Care Research
Nm: Newton-metres
NMES: Neuromuscular electrical stimulation
OCT: Optimal cutting temperature compound
PBMCs: Peripheral blood mononuclear cells
PPIE: Patient and Public Involvement and Engagement
QALYs: Quality-adjusted life years
QoL: Quality of Life
RCT: Randomised controlled trial
R&D/I: Research and Development/Innovation
REC: Research Ethics Committee
REDCap: Research Electronic Data Capture
RNA: Ribonucleic acid
RPE: Rating of perceived exertion
SAE: Serious adverse event
SARC-F: Strength, Ambulation, Rising from a chair, Stair climbing and history of Falling
SarQoL: Sarcopenia-related quality of life
SAP: Statistical analysis plan
SONG-HD: Standardised Outcomes in Nephrology—Haemodialysis
SOP: Standard operating procedure
SPPB: Short physical performance batter
STS5: Sit to stand 5
TENS: Transcutaneous electrical nerve stimulation
TMG: Trial Management Group
TSC: Trial Steering Committee
UKKA: UK Kidney Association
6MWT: Six-minute walk test
